# miR‐941 in extracellular vesicles confers anlotinib resistance via Keap1/Nrf2 axis and represents a therapeutic target in non‐small cell lung cancer

**DOI:** 10.1002/ctm2.70721

**Published:** 2026-06-15

**Authors:** Aimi Huang, Xiaoqi Li, Erpeng Wu, Menglan Hao, Weimin Wang, Jinjing Xia

**Affiliations:** ^1^ Department of Respiratory and Critical Care Medicine Shanghai Key Laboratory of Thoracic Tumor Biotherapy Shanghai Chest Hospital, Shanghai Jiao Tong University Shanghai China

**Keywords:** anlotinib resistance, extracellular vesicles, Keap1/Nrf2 pathway, miR‐941, non‐small cell lung cancer, predictive biomarker

## Abstract

**Background:**

Anlotinib is a multitargeted tyrosine kinase inhibitor for the treatment of advanced non‐small cell lung cancer (NSCLC), though its therapeutic efficacy is constrained by acquired resistance. Although elevated extracellular vesicle (EV)‐derived *miR‐941* correlates with anlotinib resistance, its functional role remains unexplored. Here, we provide evidence that EV‐delivered *miR‐941* mediates this resistant phenotype.

**Methods:**

Functional experiments (gain‐ and loss‐of‐function) in A549 and H1299 cells assessed viability, apoptosis and migration. The *miR‐941*/*Keap1* interaction was validated using dual‐luciferase assays. In vivo resistance was evaluated using wild‐type and *Keap1* 3'UTR‐mutant xenograft models. Clinical relevance and survival outcomes were analysed in tumour tissues from anlotinib‐treated patients.

**Results:**

EV‐derived *miR‐941* was significantly upregulated in anlotinib‐resistant patients. Mechanistically, *miR‐941* directly targeted the *Keap1* 3′UTR, suppressing Keap1 protein and activating the Nrf2 signalling pathway. This axis inhibited apoptosis, upregulated anti‐apoptotic proteins (Bcl‐2, Mcl‐1), and promoted malignant phenotypes in vitro. Crucially, in vivo anlotinib resistance induced by *miR‐941* overexpression was completely abrogated by mutating the *Keap1* binding site. Clinical samples confirmed elevated *miR‐941* and a molecular signature consistent with Keap1 downregulation and Nrf2 activation in resistant tumours. Furthermore, high expression of *miR‐941* was strongly associated with shorter progression‐free survival and overall survival.

**Conclusion:**

In this study, we identified EV‐derived *miR‐941* as a key driver of anlotinib resistance via the *Keap1*/Nrf2 pathway. It represents a promising non‐invasive predictive biomarker and serves as a candidate therapy target for overcoming drug‐resistant NSCLC.

**Key points:**

EV‐derived miR‐941 as a key driver of anlotinib resistance via the Keap1/Nrf2 pathway represent a promising non‐invasive predictive biomarker and a potential therapeutic target for overcoming resistance in NSCLC.

## Graphical abstract

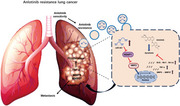

## 1 BACKGROUND

Currently, lung cancer remains the primary leading cause of death from cancer, with non‐small cell lung cancer (NSCLC) comprising about 85% of the total cases.^1^ Advances in targeted therapy and immunotherapy have markedly enhanced treatment outcomes for selected populations, especially those with druggable driver genetic mutations or elevated PD‐L1 expression.^2^, ^3^ Nevertheless, most patients eventually become resistant to these treatments, and a subset exhibit primary resistance, leading to disease progression.^4^ For this growing patient population, especially those lacking actionable driver mutations, effective late‐line therapeutic options are critically needed to extend survival in this setting.

Anlotinib, an investigational new oral multitargeted tyrosine kinase inhibitor (TKI), has emerged as a valuable therapeutic option for advanced NSCLC.^5^, ^6^ Mechanistically, this agent restricts tumour neovascularization and malignant cell expansion by concurrently antagonizing a spectrum of receptor tyrosine kinases, notably VEGFRs, FGFRs, PDGFRs and various other kinases.^7^ The pivotal ALTER‐0303 phase III trial demonstrated that anlotinib markedly prolonged progression‐free survival (PFS) and overall survival (OS) among patients with refractory advanced NSCLC, leading to its approval in China as a third‐line or later treatment.^8^ However, there are significant individual differences in the clinical benefits of anlotinib. For example, in our prior real‐world cohort study (*N* = 158), we observed significant heterogeneity in progression‐free survival (PFS), yet reliable predictive biomarkers are still lacking.^9^ The unpredictability of this therapeutic effect poses a major challenge in clinical practice.

The liquid biopsy represents a promising method for dynamic monitoring of tumour molecular profiles.^10^ Among various liquid biopsy analytes, extracellular vesicles (EVs), particularly small extracellular vesicles (sEVs), have garnered significant attention for their stability in the circulatory system and the biologically active molecules they carry, which include proteins, nucleic acids, and lipids.^11^ EVs are secreted by parentocytes and reflect their pathophysiological state; they serve as key intermediaries in intercellular signalling. The lipid‐bilayer structure of their membrane protects the internal contents from enzymatic degradation. MicroRNAs (miRNAs) carried by EVs are modulators of genetic expression and critically influence tumour initiation, progression and drug resistance.^12^, ^13^ Thus, miRNAs derived from EV are promising candidate biomarkers.

In a previous exploratory study, we identified a significant association between levels of *miR‐941* in circulating sEVs and resistance to anlotinib in patients with advanced NSCLC.^14^ While miRNAs are frequently implicated in drug resistance, their functions are highly context‐dependent, varying with tumour type, therapeutic agent and microenvironment. Notably, *miR‐941* has been previously reported to promote proliferation in breast cancer and lung adenocarcinoma, but its role in mediating resistance to a specific targeted therapy remains unexplored, particularly anlotinib. Our prior observation that patients with disease progression after anlotinib treatment exhibited significantly elevated levels of sEV‐derived *miR‐941* prompted us to investigate whether this miRNA functionally drives anlotinib resistance.

## 2 METHODS

### 2.1 Patient cohorts and data collection

The present research was performed in compliance with the Declaration of Helsinki and was granted approval via the Institutional Ethics Review Board of Shanghai Chest Hospital (Approval No. IS22010). Signed consents have been received for all participants.

For the validation of the *miR‐941*/*Keap1*/*Nrf2* signalling pathway, fresh tumour biopsy tissue samples were collected from 14 NSCLC patients who received anlotinib treatment at our institution. Patients were categorized as anlotinib‐sensitive or anlotinib‐resistant (exhibiting progressive disease) based on their best clinical response. Tissue specimens are rapidly frozen in cryogenic nitrogen and maintained at ‐80°C. A comprehensive summary of clinicopathological characteristics for the 14 patients included in this study is provided in Table S1.

### 2.2 Cell lines and culture

The A549 cells (ATCC) were cultivated in DMEM high glucose medium (Hyclone, SH30243.FS) containing 10% FBS (Thermo, Cat#10099141C), 100 U/mL penicillin and 100 µg/mL streptomycin (Thermo, Cat#15140122). H1299 cells (ATCC) were cultured in RPMI‐1640 medium (Gibco, Cat#C11875500BT). All cells were cultured at 37°C in a humidity‐controlled environment with 5% CO_2_. For in vitro treatments, the following reagents were used: anlotinib (MCE, Cat#HY‐101416), ML385 (MCE, Cat#HY‐100523; specific Nrf2 inhibitor), the TNF‐α neutralizing antibody infliximab (MCE, Cat#HY‐P9970), and the pan‐caspase inhibitor Z‐VAD‐FMK (MCE, Cat#HY‐16658B, 20 µM, 24 h).

### 2.3 Extracellular vesicle isolation and characterization

Peripheral blood samples from patients with progressive disease (PD) following anlotinib treatment and healthy controls were collected in EDTA‐coated tubes. To isolate EVs, plasma samples were first spun down at 3000 ×g (4°C, 10 min). Cell fragments and macrovesicles were then cleared by spinning the mixture at 10 000 ×g for 30 min. Following this, the remaining supernatant was ultracentrifuged at 100 000 ×g for 70 min (Thermo, LYNX6000) to precipitate the sEV fraction.

For cell cultured EVs, culture A549 cells for 48 h in media containing 10% EV‐depleted FBS (fabricated by centrifugation at 100 000 ×g for 18 h). Conditioned medium was collected and subjected to the same differential ultracentrifugation protocol as described above. The concentration of the EV protein was measured with the BCA‐based assay kit (Sangon Biotech, Cat#C503021). Validation of EV purity was performed by WB analysis of EV‐positive markers (CD63, TSG101, CD9 and CD81), as described in Section 2.6.2.

### 2.4 Stable cell line construction and transient transfection

#### 2.4.1 miRNA mimics, inhibitors and transfection

All synthetic oligonucleotides—including the *miR‐941* mimic, its specific inhibitor (anti‐*miR‐941*), and the corresponding scrambled sequences (NC)—were commercially manufactured by GenePharma. For intracellular delivery, A549 and H1299 populations were grown to approximately 65% density in standard six‐well formats before being exposed to a 50 nM oligonucleotide mixture, strictly adhering to the established commercial protocol. The transfection complexes were removed and replaced with standard growth media roughly 6–8 h post‐exposure. Subsequent downstream functional analyses were conducted using cells collected at the 48‐h mark.

#### 2.4.2 Lentivirus‐mediated stable cell line construction

We constructed lentiviral vectors for miR‐941 upregulation and control, and performed packaging in 293T cells using the psPAX2 and pMD2.G plasmid vectors (provided by Dr. Daming Gao in SIBCB). Collect the virus‐containing culture supernatant 48–72 h after transfection. After filtration, use it to infect A549 cells in the absence of 4 µg/mL polyburen. Subsequently, screen for stable clones using 2 µg/mL procaine.

#### 2.4.3 Genome editing via crispr/cas9 and specificity validation

Regarding CRISPR/Cas9‐mediated gene elimination, sgRNAs targeting *Keap1* and *Nrf2* (sequences are listed in Table S2) were designed and cloned into the lentiCRISPR v2 vector (provided by Dr. Liming Sun in SIBCB). Lentiviruses were produced and used to infect A549 cells. Single‐cell clones were isolated, expanded and the specificity of the target knockout was validated by Western blot and genomic sequencing.

#### 2.4.4 Keap1 3′utr mutagenesis

For *Keap1* 3′UTR mutation, a homologous recombination template was designed and introduced using the pAAVS1‐RFP‐DNR‐KEAP1‐Mut plasmid, followed by selection and verification.

#### 2.4.5 Extracellular vesicle co‐culture assay

To evaluate the functional transfer of EVs, recipient A549 were plated in 6‐well plates. When cell confluence reached 60%–70%, cells were co‐cultured with isolated sEVs (normalized to 20 µg/mL of total EV protein) in a medium containing with 10% EV‐depleted FBS for 48 hours prior to anlotinib treatment or subsequent functional assays.

### 2.5 In vitro functional assays

#### 2.5.1 Cell viability and apoptosis assays

Cellular viability was assessed using the CellTiter‐Glo Luminescence Assay Kit (Promega, Cat#G7572), with the procedure strictly following the supplier's instructions. After seeding cells into 96‐well plates and treating them with the drug for a specified duration, the luminescence was measured. To assess apoptosis, we measured the expression of cleaved *caspase‐3* and *PARP* via WB analysis and further confirmed the results using a caspase‐3 activity assay kit (Beyotime, Cat#C1115). In addition, Annexin V‐FITC/propidium iodide (PI) double‐staining was conducted to quantify apoptotic cell populations. Briefly, collect the cells after treatment, wash them with cold PBS, and then suspend them in 100 µL of staining buffer, which contains 5 µL Annexin V‐FITC and 5 µL PI (MCE, Cat#HY‐D0815). After incubating for 15 min at ambient temperatures under dark conditions, the samples were processed on a BD FACSCanto II flow cytometer. The data were analysed using FlowJo software (v10.9.0). Each experiment included at least three distinct biological replicate sets.

#### 2.5.2 Cell migration and invasion assay

Evaluate cell migration through scratch wound assays. Seed cells in 6‐well plates and culture until the cells form a confluent monolayer. Create scratches using a sterilized pipette tip, then wash the cells to remove debris. Take images at 0, 24 and 48 h, and quantify the migratory distance via ImageJ software (version 1.x). Wound closure percentage was calculated as the ratio of the migrated area to the initial wound area.

To further evaluate cell motility, Transwell migration assays were performed. Briefly, A549 cells (5 × 10^4^) were cultured in serum‐free DMEM and inoculated into the upper chambers of Transwell filters (Corning, Cat#33422; pore size 8 µm). DMEM containing 10% FBS was added to the lower chambers to serve as the chemotactic factor. Migrating cells on the lower surface were fixated with 4% paraformaldehyde, counterstained with 0.1% crystal violet, and quantified under an optical microscope in three random fields per insert.

For Transwell invasion assays, coat the upper chamber with a Matrigel matrix (Corning, Cat#354234) pre‐diluted 1:8 in DMEM without serum. The Matrigel‐coated inserts were cultured at 37°C for 2 h to ensure full gelation before cell seeding. A549 cells (5 × 10^4^) were resuspended in serum‐free DMEM and added to the pre‐coated upper chambers. The remaining procedures (chemoattractant setup, cell fixation, staining and counting) were identical to those used for Transwell migration assays.

At least three biological replicates were used for all experiments.

### 2.6 Molecular and biochemical analyses

#### 2.6.1 RNA extraction and qRT‐PCR

Total cellular RNA was isolated using a commercial extraction reagent (Thermo, Cat#15596018CN). To quantify mRNA transcripts, 1 µg of the purified RNA served as a template for synthesizing first‐strand cDNA via the Vazyme reverse transcription kit (Cat#R333‐01), with all specific primer sequences detailed in Table 1. To specifically evaluate miRNA expression, cDNA was synthesized via targeted stem‐loop primers utilizing the TaqMan MicroRNA Reverse Transcription Kit (Cat#4366596), strictly adhering to the developer's recommended protocols. Subsequent real‐time amplification was executed on an Applied Biosystems platform, employing the ChamQ SYBR Green formulation (Vazyme, Cat#Q411‐03). The comparative cycle threshold (2^−ΔΔCt^) approach was applied for data quantification, relying on *U6* (for miRNAs) or *GAPDH* (for mRNAs) as endogenous reference markers.

**TABLE 1:** List of q‐PCR primer.

Gene		Sequence
*Bcl2*	F	GGTGGGGTCATGTGTGTGG
	R	CGGTTCAGGTACTCAGTCATCC
*TNF‐α*	F	CCTCTCTCTAATCAGCCCTCTG
	R	GAGGACCTGGGAGTAGATGAG
*Bcl‐xl*	F	CCCAGAGTTTGAGCCGAGTG
	R	CCCATCCCTTCGTCGTCCT
*HSP70*	F	TTTGAGGGCATCGACTTCTACA
	R	CCAGGACCAGGTCGTGAATC
*miR‐941*	F	CACCCGGCTGTGTGCACATGTGC
	R	GCACATGTGCACACAGCCGGGTG
*KEAP1*	F	CTGGAGGATCATACCAAGCAGG
	R	GGATACCCTCAATGGACACCAC
*NRF2*	F	TCAGCGACGGAAAGAGTATGA
	R	CCACTGGTTTCTGACTGGATGT
*GAPDH*	F	GGAGCGAGATCCCTCCAAAAT
	R	GGCTGTTGTCATACTTCTCATGG
*ACTIN*	F	CATGTACGTTGCTATCCAGGC
	R	CTCCTTAATGTCACGCACGAT
*MMP3*	F	AGTCTTCCAATCCTACTGTTGCT
	R	TCCCCGTCACCTCCAATCC
*MMP7*	F	GAGTGAGCTACAGTGGGAACA
	R	CTATGACGCGGGAGTTTAACAT
*MMP9*	F	TGTACCGCTATGGTTACACTCG
	R	GGCAGGGACAGTTGCTTCT
*MMP27*	F	GGGCCAGTATGGCTACACC
	R	CAAGGGACCATCAAAATAGCGA
*Mcl1*	F	TGCTTCGGAAACTGGACATCA
	R	TAGCCACAAAGGCACCAAAAG
*Nqo1*	F	CCTGCCATTCTGAAAGGCTGGT
	R	GTGGTGATGGAAAGCACTGCCT
*Gclc*	F	GGAAGTGGATGTGGACACCAGA
	R	GCTTGTAGTCAGGATGGTTTGCG

#### 2.6.2 Western blotting

Total protein pools were extracted by disrupting cells in a commercially available RIPA lysis solution (Beyotime, Cat#P0013B) enriched with a dual inhibitor cocktail (Share‐bio, Cat#SB‐WB017) to prevent degradation. Following yield quantification via a standard Bradford protocol (Sangon, Cat#C503041), uniform quantities of protein lysates underwent electrophoretic separation across SDS‐polyacrylamide gels. These resolved bands were electrically transferred onto polyvinylidene fluoride (PVDF) membranes. Non‐specific binding was minimized by a 1‐h immersion in 5% defatted milk, preparing the blots for specific primary antibody probing at 4°C overnight. Signal visualization was achieved after applying an appropriate horseradish peroxidase‐linked secondary antibody. Comprehensive information regarding the specific antibodies and their respective dilution protocols is provided in Table 2.

**TABLE 2:** List of antibodies.

Name of antibody	Source	Batch number	Dilution ratio
Anti‐Bid Cleavage Site antibody	Abcam	Cat#ab10640‐50ul	1:1000
Anti‐Caspase‐3 antibody [EPR18297]	Abcam	Cat#ab184787‐40ul	1:1000
Anti‐PARP Antibody	CST	Cat#9542	1:1000
Anti‐MMP3 antibody [EP1186Y]	Abcam	Cat#ab52915‐40u	1:1000
Anti‐KEAP1 antibody	CST	Cat#8047	1:1000
Anti‐NRF2 antibody	CST	Cat#12721	1:1000
Anti‐MMP‐9 antibody human specific	CST	Cat#3852	1:1000
Anti‐MMP‐9 antibody mouse specific	CST	Cat#24317	1:1000
Anti‐MMP‐27 antibody	Abcam	Cat#ab150415	1:1000
Anti‐Bcl2 antibody	CST	Cat#3498	1:1000
Anti‐Mcl‐1 antibody	CST	Cat#5453	1:1000
Anti‐Bcl‐xL antibody	CST	Cat#2764	1:1000
BID Antibody (Mouse Specific)	CST	Cat#2003S	1:1000
E‐Cadherin (4A2) Mouse mAb	CST	Cat#14472S	1:1000
GAPDH (D4C6R) Mouse mAb	CST	Cat#97166S	1:1000
β‐Actin (8H10D10) Mouse mAb	CST	Cat#3700T	1:1000
Anti‐Histone H3 antibody	Proteintech	Cat#68345‐1‐Ig	1:1000
GOATaRABBIT IRDye 800CW, 0.5 mg	Licor	Cat#926‐32211	1:1000
Anti‐CD63 antibody	CST	Cat# 52090	1:1000
Anti‐TSG101 antibody	CST	Cat# 72312	1:1000
Anti‐CD9	CST	Cat# 13174	1:1000
Anti‐CD81	CST	Cat# 56039	1:1000

#### 2.6.3 Nuclear‐cytoplasmic fractionation

Extract nuclear and cytoplasmic proteins following the instructions provided by the manufacturer (Beyotime, Cat#P0027). The brief procedure is as follows: Wash A549 and H1299 cells with cold PBS, resuspend them in the cytoplasmic extraction buffer, and gently vortex on ice for 15 min. Centrifuge at 12 000 ×g for 10 min at 4°C, then collect the pellet supernatant (cytoplasmic fraction). Resuspend the precipitate in a nuclear extraction reagent and centrifuge to obtain the nuclear fraction. Albumin content was measured using the Bradford method. Histone H3 (CST, Cat#9715, 1:5000) was used as a nuclear loading control, and GAPDH (CST, Cat#2118, 1:5000) was used as a cytoplasmic loading control.

#### 2.6.4 Immunofluorescence staining and confocal microscopy

Seed A549 cells onto glass‐coated in 24‐well plates and transfected with *miR‐941* mimic or negative control as described above. 48 h after transfection, the cells were rinsed with PBS and fixated with 4% formaldehyde at room temperature for 15 min. After permeabilization with a solution of 0.2% Triton X‐100 in PBS for 20 min, the cells were incubated with 5% bovine serum albumin at room temperature for 1 h. Subsequently, the cells were incubated overnight at 4°C with anti‐Nrf2 primary antibody (CST, Cat#12721, diluted 1:200), then add the goat anti‐rabbit IgG secondary antibody (Thermo, Cat#A32731) and incubate for 1 h. Cell nuclei were counter‐stained for 5 min with DAPI (MCE, Cat#HY‐D2868, 1:1000). Coverslips were fixed to slides using an anti‐fade mounting medium (MCE, Cat#HY‐K1042). Imaging was acquired via Leica TCS SP8 confocal laser scanning microscopy. ImageJ software (NIH) was used to perform quantitative analysis of nuclear and cytoplasmic fluorescence intensities, and the nuclear‐to‐cytoplasmic ratio of Nrf2 signalling was determined based on at least 20 cells per group.

#### 2.6.5 Enzyme‐linked immunosorbent assay (ELISA)

TNF‐α concentrations were then quantified using a human TNF‐α ELISA kit (Beyotime, Cat#PT512) following the supplier's instructions. Measure the absorbance at 450 nm, with 570 nm as the reference wavelength (BioTek, Cat#SYNERGY H1). All samples were assayed in duplicate, and cytokine levels were quantified using the standard curve interpolation method.

#### 2.6.6 Dual‐luciferase reporter assay

The wild‐type (WT) or mutant (MUT) 3′UTR sequence of *Keap1* was cloned into the pEZX vector (GeneCopoeia, Cat#zt531). Using PEI transfection reagent, 293T cells were cotransfected with the report plasmid, the pRL‐TK sea‐kidney luciferase control vector (NovoPro, Cat#V011444), and either the *miR‐941* mimic or NC (Servicebio, Cat#G1802). Forty‐eight hours after transfection, luciferase activity was quantified on a microplate reader (BioTek, Cat#SYNERGY H1) using the Dual Luciferase Reporter Assay System (Vazyme, Cat#DL101‐01).

### 2.7 Animal studies

The animal testing complies with the requirements of the Committee on Animal Care and Use. Purchase female BALB/c nude mice aged 6–8 weeks from GemPharmatech and maintain them under conditions free of specific pathogens. Inject A549 cells (3 × 10^6^) subcutaneously into the ventral side of mice. When tumour volumes reached approximately 100 mm^3^, mice were randomly assigned to treatment groups. Anlotinib (2 mg/kg) or vehicle was administered via intraperitoneal injection every 3 days. Measure the tumour volume every three days and calculate it as follows: V = 0.5 × length × width^2^. Mice were euthanized after 18 days, and tumours were harvested for further analysis.

### 2.8 Statistical analysis

All experiments were performed with at least three independent biological replicates. Statistical analyses were carried out with software of GraphPad Prism 8.0 and Excel. Results are expressed as mean ± SD, except for immunofluorescence analysis and clinical sample comparisons where mean ± SEM is indicated in the corresponding figure legends. The two‐group comparisons were performed using a two‐sided unpaired Student's *t*‐test. For multi‐group comparisons, as indicated in the legend, a one‐way ANOVA was first conducted, followed by a Dunnet's test or a Tukey's test. For non‐normally distributed data in clinical sample analyses, the Mann‐Whitney *U* test was used. Based on the median expression level of miR‐941 in tumour tissue, patients were stratified into high‐expression and low‐expression groups. A *p *< 0.05 was deemed statistically significant.

## 3 RESULTS

### 3.1 *miR‐941* Promotes anlotinib resistance in lung cancer cells

**FIGURE 1:**
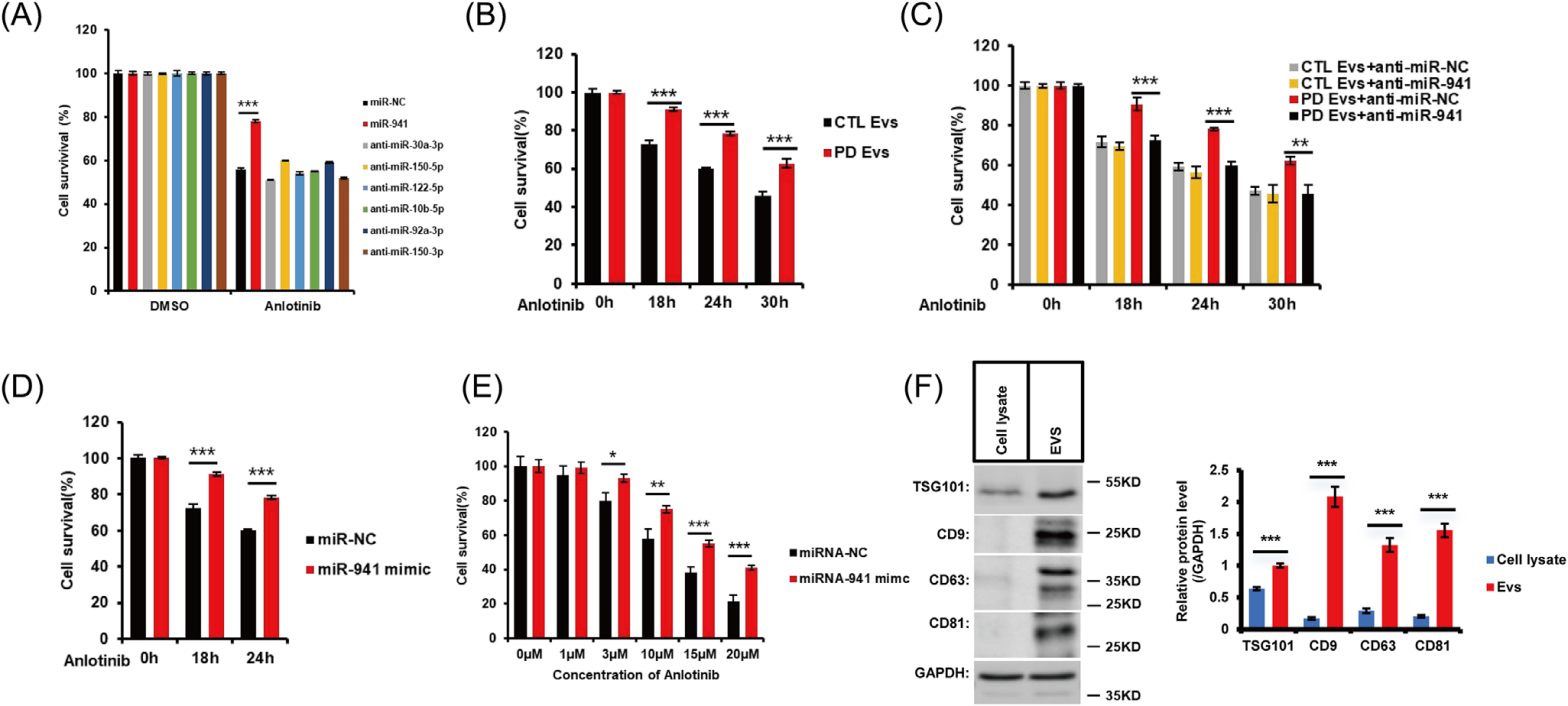
miR‐941 functionally drives anlotinib resistance in lung cancer. (A) Cell viability of A549 cells transfected with the indicated miRNAs and treated with anlotinib (10 µM). (B) Cell viability of A549 cells co‐cultured with extracellular vesicles (EVs) from patients with progressive disease (PD) or healthy controls (CTL), followed by anlotinib treatment (10 µM) for the indicated times. (C) Inhibition of miR‐941 reverses EV‐mediated anlotinib resistance. A549 cells were pre‐transfected with anti‐miR‐941 or a negative control (NC) before co‐culture with PD‐derived EVs and subsequent anlotinib treatment. (D) Overexpression of miR‐941 mimic confers resistance to anlotinib over time. A549 cells transfected with miR‐941 mimic or NC were treated with anlotinib (10 µM) for the indicated durations. (E) Overexpression of miR‐941 mimic increases the IC_50_ of anlotinib. Transfected A549 cells were treated with varying concentrations of anlotinib for 24 h. (F) Western blot analysis of EV markers CD63, TSG101, CD9 and CD81 in EVs and cell lysate. Error bars represent standard deviation (*n* = 3). *p*‐values indicated were calculated by Student's *t*‐test (unpaired). ns: *p* > 0.05, *p < 0.05; **p < 0.01; ***p < 0.001.

To investigate the functional role of *miR‐941*, which was previously identified as elevated in anlotinib‐resistant patients, we conducted gain‐ and loss‐of‐function experiments in A549 lung cancer cells. Cell viability assays revealed that overexpression of *miR‐941* enhanced cellular tolerance to anlotinib, whereas modulation of other miRNAs did not produce such effects (Figure 1A).

We next examined whether sEVs derived from patients with PD could confer anlotinib resistance. Treatment of A549 cells with sEVs isolated from PD patients significantly increased resistance to anlotinib compared to sEVs from healthy controls (Figure 1B). Importantly, this resistance‐conferring effect was markedly attenuated when cells were pre‐transfected with anti‐*miR‐941* to neutralize *miR‐941* delivered by the sEVs (Figure 1C).

To further confirm the direct role of *miR‐941*, we transiently transfected A549 cells with a *miR‐941* mimic. Compared to control cells, *miR‐941*–overexpressing cells exhibited higher viability and increased IC_50_ values following treatment with anlotinib at various time points and concentrations (Figure 1D, E). Furthermore, WB analysis verified the purity of the isolated EVs, which was confirmed by the presence of the EV markers CD63, TSG101, CD9 and CD81 (Figure 1F).

All things considered, these results demonstrate that *miR‐941* is a key functional miRNA mediating anlotinib resistance in lung cancer cells. Consistent findings were also observed in the H1299 NSCLC cell line, confirming that *miR‐941* confers anlotinib resistance across genetically distinct lung cancer cells (Figure S1A).

### 3.2 *miR‐941* Directly targets *Keap1* to activate the *Nrf2* signalling pathway

**FIGURE 2:**
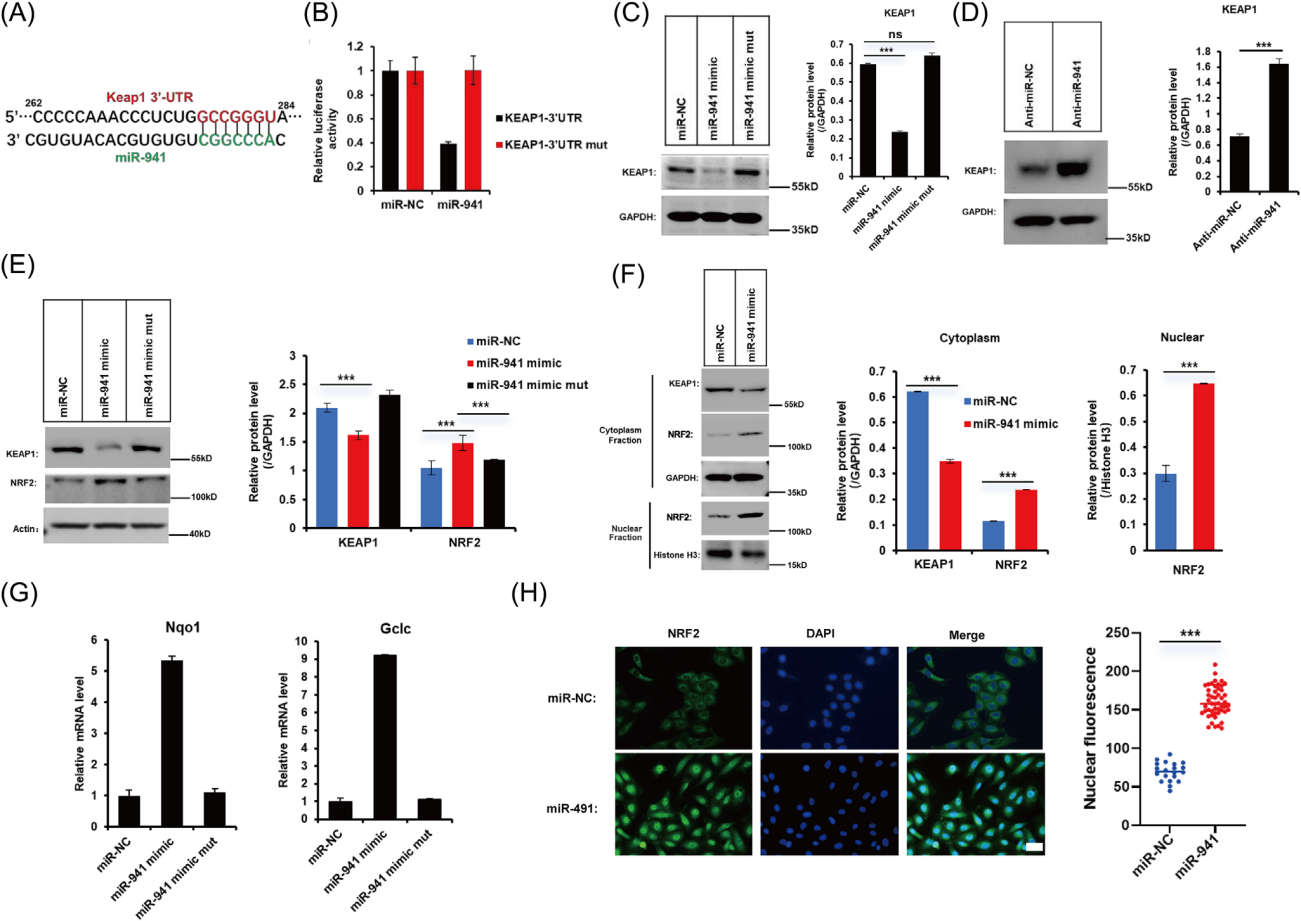
miR‐941 directly targets *Keap1* to activate the *Nrf2* signalling pathway. (A) Schematic of the predicted miR‐941 binding site in the 3′UTR of *Keap1* mRNA. (B) Dual‐luciferase reporter assay in 293T cells co‐transfected with a reporter containing wild‐type (WT) or mutant (MUT) *Keap1* 3′UTR and miR‐941 mimic or NC. (C and D) Western blot analysis of Keap1 protein levels in A549 cells following transfection with (C) miR‐941 mimic or (D) anti‐miR‐941. (E) Western blot analysis of Keap1 and Nrf2 protein levels in A549 cells transfected with miR‐941 mimic, a mutated mimic (miR‐941 mut), or NC. (F) miR‐941 promotes nuclear translocation of Nrf2. Western blot analysis of Nrf2 levels in cytoplasmic and nuclear fractions of A549 cells after miR‐941 mimic transfection. Histone H3 (nuclear) and GAPDH (cytoplasmic) were used as loading controls for nuclear and cytoplasmic fractions, respectively. Error bars represent standard deviation (*n *= 3). *p*‐values indicated were calculated by Student's *t*‐test (unpaired). ns: *p* > 0.05, *p < 0.05; **p < 0.01; ***p < 0.001. (G) RT‐qPCR analysis of *Nrf2* target genes (*Nqo1*, *Gclc*) in A549 cells transfected with miR‐941 mimic, miR‐941 mut, or NC. (H) Representative immunofluorescence images and quantitative analysis of Nrf2 subcellular localization in A549 cells transfected with miR‐941 mimic or NC. Scale bar, 20 µm. Data represent mean ± SEM. (miR‐NC, *n* = 20; miR‐941, *n* = 50). Statistical differences were tested using ANOVA followed by Tukey test. ***p < 0.001.

In order to clarify the molecular mechanism through which *miR‐941* mediates its effects, we performed bioinformatic analysis and detected a potential binding site for *miR‐941* within the 3′UTR of *Keap1* (Figure 2A). We validated this direct interaction by constructing luciferase reporter plasmids harbouring either the WT or MUT *Keap1* 3′UTR. The dual‐luciferase assay revealed that *miR‐941* markedly suppressed the luciferase activity of the WT reporter plasmid, while having no effect on the MUT construct (Figure 2B).

We further examined the regulatory effect of *miR‐941* on endogenous Keap1 protein expression. Overexpression of *miR‐941* in A549 cells led to a marked downregulation of Keap1 protein, whereas inhibition of endogenous *miR‐941* resulted in increased Keap1 levels (Figure 2C,D). Since Keap1 is a key negative regulator of Nrf2, we next assessed Nrf2 expression. As expected, overexpression of *miR‐941*, which induced the knockdown of *Keap1*, led to a significant accumulation of total Nrf2 protein (Figure 2E).

To confirm functional activation of the *Nrf2* pathway, we assessed its subcellular localization. Nucleocytoplasmic separation experiments indicate that overexpression of *miR‐941* promoted the nuclear translocation of Nrf2 (Figure 2F), a hallmark of its activation as a transcription factor. Consistent with this, RT‐qPCR analysis showed that the mRNA levels of classical *Nrf2* downstream target genes, *Nqo1* and *Gclc*, were significantly upregulated (Figure 2G). To further validate the enhanced nuclear accumulation of Nrf2, immunofluorescence staining was performed. As shown in Figure 2H, compared with control, miR‐941 overexpression markedly increased nuclear Nrf2 signalling; quantification of fluorescence intensity confirmed that the enrichment of Nrf2 in the nuclear compartment was statistically significant.

Taken together, the results demonstrate that *miR‐941* directly targets *Keap1*, thereby relieving its repression of *Nrf2* and consequently activating the *Nrf2* signalling pathway. These regulative axes were further validated in H1299 cells, where *miR‐941* overexpression similarly downregulated Keap1, upregulated Nrf2 and promoted Nrf2 nuclear translocation (Figure S1B,C).

### 3.3 The anti‐apoptotic function of *miR‐941* is dependent on the *Keap1*/*Nrf2* axis

**FIGURE 3:**
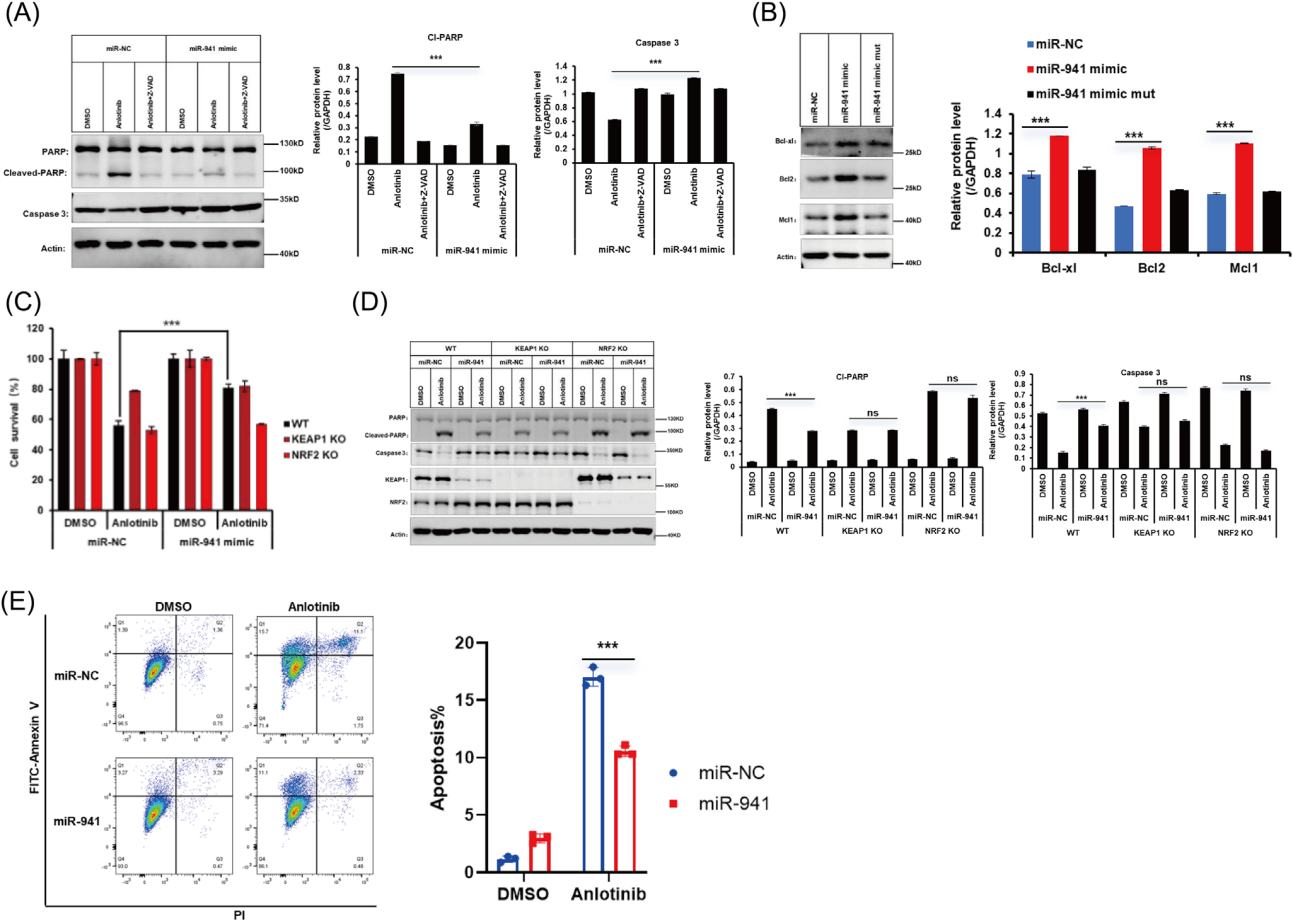
The anti‐apoptotic effect of miR‐941 is dependent on the *Keap1*/*Nrf2* axis. (A) Western blot analysis showing that miR‐941 overexpression suppresses anlotinib‐induced PARP, Cleaved‐PARP and Caspase 3 in A549 cells. Z‐VAD‐FMK (20 µM, 24 h) was used as a pan‐caspase inhibitor control (shown in the indicated lanes). (B) Western blot analysis of anti‐apoptotic proteins (Mcl‐1, Bcl‐2, Bcl‐xL) in A549 cells transfected with miR‐941 mimic or NC. (C) Knockout of *Keap1* or *Nrf2* abolishes the pro‐survival effect of miR‐941. Cell viability of wild‐type (WT), *Keap1*‐KO, and *Nrf2*‐KO A549 cells transfected with miR‐941 mimic or NC and treated with anlotinib. (D) Western blot analysis confirming that the anti‐apoptotic effect of miR‐941 is lost in *Keap1*‐KO and *Nrf2*‐KO cells, as indicated by *PARP* cleavage. (E) Representative flow cytometry plots and quantification of apoptotic cells (Annexin V‐FITC‐positive) in A549 cells. Error bars represent standard deviation (*n* = 3). *p*‐values indicated were calculated by Student's *t*‐test (unpaired). ***p < 0.001.

One of the primary antitumour mechanisms of anlotinib is the induction of apoptosis. Our findings indicate that overexpression of *miR‐941* markedly inhibited anlotinib‐induced cleavage of *PARP*, a key apoptotic marker (Figure 3A). Mechanistically, upregulation of *miR‐941* promoted the expression of critical anti‐apoptotic proteins, including Bcl‐2, Mcl‐1 and Bcl‐xL (Figure 3B).

To establish causality and rule out potential compensatory pathway activation, we performed rescue experiments using *Keap1*‐knockout (KO) or *Nrf2*‐KO A549 cells. Cell viability assays revealed that while *miR‐941* significantly enhanced cell survival in wild‐type (WT) cells, this pro‐survival effect was largely abolished in both *Keap1*‐KO and *Nrf2*‐KO cells (Figure 3C). WB analysis confirmed that, at the molecular level, *miR‐941* was no longer able to suppress anlotinib‐induced *PARP* cleavage in *Keap1*/*Nrf2*‐KO cells (Figure 3D). To further quantify the anti‐apoptotic effect of *miR‐941*, we performed Annexin V‐FITC/PI staining, which was followed by flow cytometric analysis. As shown in Figure 3E, anlotinib pretreatment markedly increased the proportion of apoptotic cells (Annexin V‐positive) in control A549 cells, whereas this effect was markedly attenuated in cells overexpressing *miR‐941*.

Together, these data strongly demonstrate that *miR‐941* confers anlotinib resistance by activating the *Keap1*–*Nrf2* pathway, leading to the upregulation of anti‐apoptotic proteins and subsequent inhibition of apoptosis. Consistent with these observations, *miR‐941* overexpression in H1299 cells significantly reduced anlotinib‐induced apoptosis and suppressed cleavage of PARP and Caspase‐3 (Figure S2).

### 3.4 The *miR‐941*/*Keap1*/*Nrf2* axis enhances the malignant potential of lung cancer cells

**FIGURE 4:**
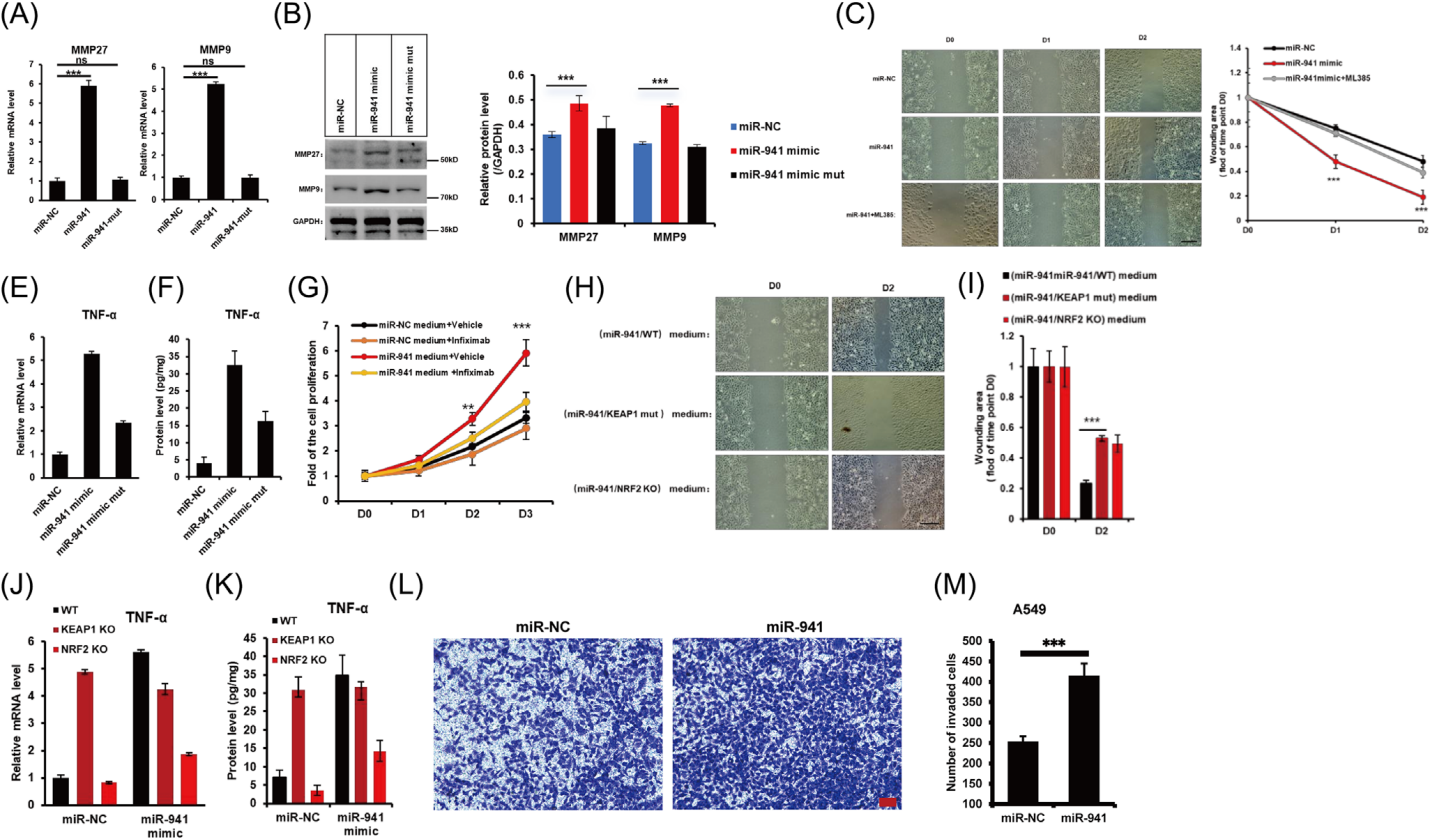
The miR‐941/*Keap1*/*Nrf2* axis promotes malignant phenotypes of proliferation and migration. (A, B) *miR‐941* upregulates MMP9 and MMP27 at the mRNA (A) and protein (B) levels in A549 cells. (C and D) *miR‐941* promotes cell migration in an Nrf2‐dependent manner. (C) Representative wound healing images in A549 cells transfected with *miR‐941* mimic or NC, with or without ML385 (5 µM). Scale bar, 200 µm. (D) Quantification of wound closure. (E and F) *miR‐941* upregulates TNF‐α mRNA (E) and protein (F) levels. (G) Pro‐proliferative effect of *miR‐941* is mediated by secreted TNF‐α. Cell viability of A549 cells treated with conditioned medium from *miR‐941*‐overexpressing cells, with or without Infliximab. (H and I) Pro‐migratory effect requires intact Keap1/Nrf2 axis. (H) The wound healing assay of cells treated with miR‐941/WT medium, miR‐941/KEAP1 mut medium and miR‐941/NRF2 KO medium. Scale bar, 200 µm. (I) Quantification of wound closure. (J and K) TNF‐α upregulation depends on Keap1/Nrf2 axis, measured by RT‐qPCR (J) and ELISA (K) in Keap1‐mutant and Nrf2‐KO cells. (L and M) Transwell invasion assays in A549 cells transfected with *miR‐941* mimic or NC. (L) Representative images. Scale bar, 100 µm. (M) Quantification of migrated cells per field. Error bars represent standard deviation (*n* = 3). *p*‐values indicated were calculated by Student's *t*‐test (unpaired). ns: *p *> 0.05, *p < 0.05; **p < 0.01; ***p < 0.001.

Beyond drug resistance, we sought to determine if *miR‐941* also enhances other malignant behaviours. Indeed, overexpression of *miR‐941* promoted cell migration, as evidenced by increased expression of matrix metalloproteinases *MMP9* and *MMP27* and accelerated wound closure (Figure 4A–D). Concurrently, *miR‐941* enhanced cell proliferation, an effect associated with the upregulation of the pro‐proliferative cytokine *TNF‐α* (Figure 4E–G).

To ascertain whether these effects were causally mediated by the *Keap1*/*Nrf2* pathway and to exclude contributions from alternative signalling routes, we used cells with precise genetic modifications of the axis. Crucially, in cells harbouring a mutation in the *miR‐941* binding site of the *Keap1* 3'UTR, which prevents *miR‐941*‐mediated repression, or in *Nrf2*‐knockout cells, the ability of miR‐941 to promote migration and induce TNF‐α expression was completely abrogated (Figure 4H–K). As shown in Figure 4L,M, compared with control, miR‐941 overexpression markedly enhanced the invasive capacity of A549 cells. This demonstrates that an intact *miR‐941*/*Keap1*/*Nrf2* signalling axis is indispensable for these pro‐malignant functions.

These genetic findings were further corroborated by pharmacological approaches. Treatment with ML385, a specific *Nrf2* inhibitor, effectively abrogated the pro‐migratory effect of *miR‐941* (Figure 4C,D). Furthermore, to confirm that *TNF‐α* was a key downstream effector of the proliferative advantage, we showed that neutralizing secreted *TNF‐α* with an antibody significantly attenuated the increase in cell viability induced by *miR‐941* (Figure 4G).

Overall, these results indicate that the miR‐941/Keap1/Nrf2 regulatory axis not only mediates anlotinib resistance but also concurrently enhances the malignant potential of lung cancer cells by promoting migration and proliferation. The pro‐migratory and pro‐invasive functions of the *miR‐941*/Keap1/Nrf2 axis were also recapitulated in H1299 cells, as evidenced by enhanced wound closure, increased Transwell invasion, and upregulation of MMP9, MMP27, Bcl‐xL, Bcl‐2, and Mcl‐1 (Figures S3 and S4).

### 3.5 Targeting *Keap1* is crucial for *miR‐941*–mediated anlotinib resistance in vivo

**FIGURE 5:**
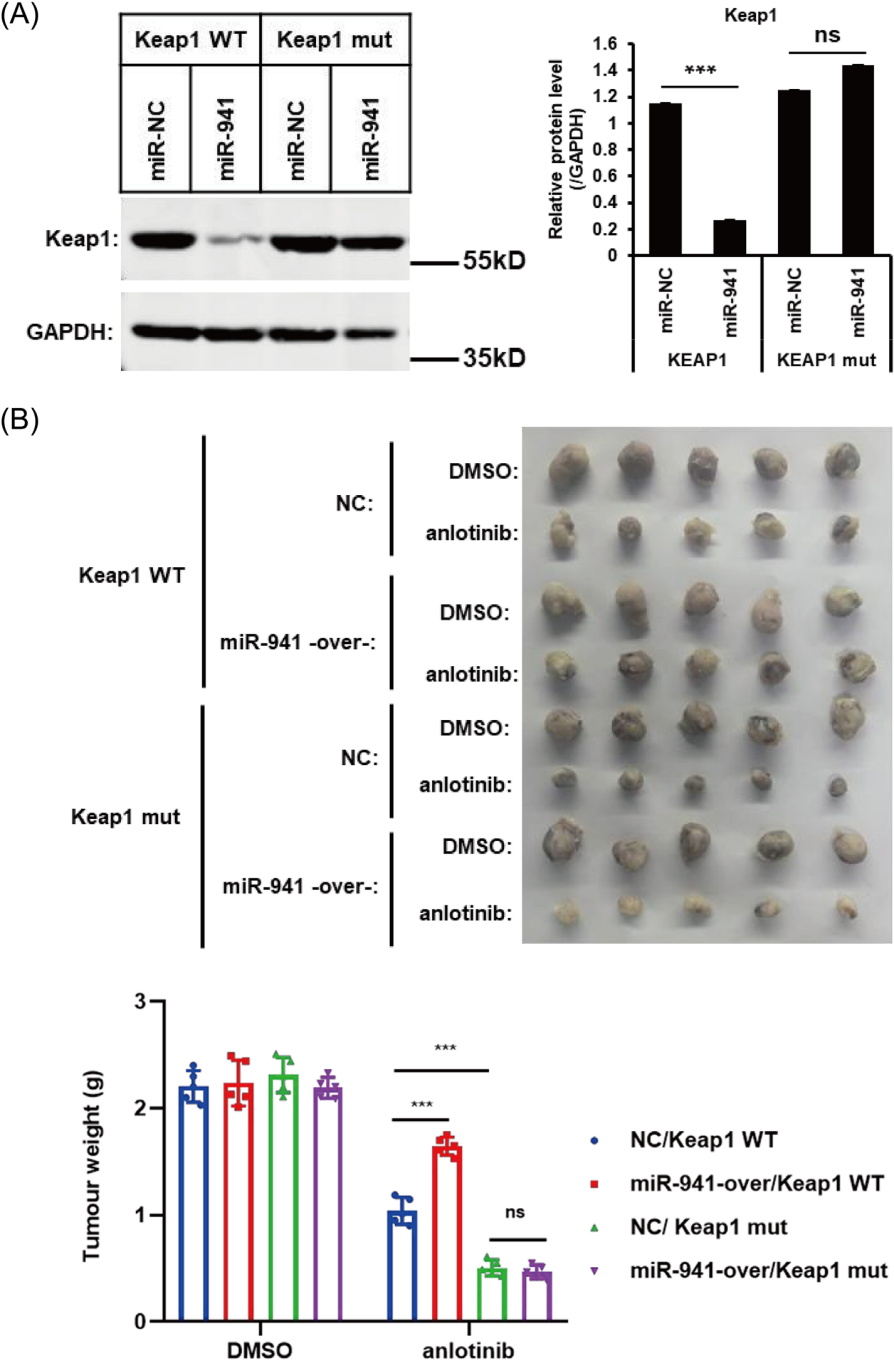
Targeting *Keap1* is essential for miR‐941‐driven anlotinib resistance in vivo. (A) Western blot validating that mutation of the miR‐941 binding site in the *Keap1* 3'UTR prevents miR‐941‐mediated downregulation of Keap1 protein. (B) Representative images of subcutaneous xenograft tumours derived from A549 cells (WT or Keap1‐mut) stably overexpressing either miR‐941 or a control vector, following treatment with anlotinib or DMSO. Below, quantification of tumour weights at the end of treatment (*n* = 5 mice per group). Error bars represent standard deviation (*n* = 3). *p*‐values indicated were calculated by Student's *t*‐test (unpaired). ns: *p *> 0.05, *p < 0.05; **p < 0.01; ***p < 0.001.

To validate our findings in vivo, we established A549 cell lines with a mutation in the endogenous *miR‐941* binding site of the *KEAP1* 3′UTR (A549‐KEAP1‐mutant), alongside their wild‐type counterparts (A549‐WT). Western blot analysis confirmed that while *miR‐941* overexpression markedly reduced Keap1 protein levels in wild‐type cells, this downregulation was completely prevented in Keap1‐mutant cells, validating the functional disruption of the *miR‐941*–Keap1 interaction (Figure 5A). Then, we stably overexpressed *miR‐941* in both cell lines to generate four experimental groups for our xenograft model. These cells were subcutaneously inoculated into nude mice to generate xenograft models. After tumour formation, mice were treated with either anlotinib or vehicle control.

Following treatment, tumours derived from WT cells overexpressing miR‐941 exhibited significant resistance to anlotinib, as evidenced by markedly larger tumour volumes compared to controls. In contrast, tumours from Keap1‐mutant cells, even those overexpressing miR‐941, remained sensitive to anlotinib, with their growth effectively suppressed by the treatment (Figure 5B).

These results provide strong in vivo evidence that the ability of *miR‐941* to drive anlotinib resistance is strictly dependent on its direct targeting of *Keap1*.

### 3.6 The *miR‐941*/*Keap1*/*Nrf2* signalling signature is validated in anlotinib‐resistant patient tumours

**FIGURE 6:**
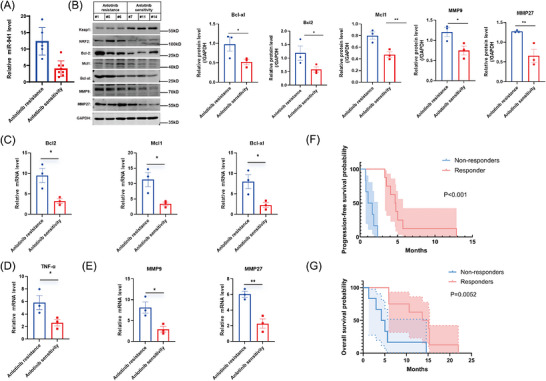
The miR‐941/*Keap1*/*Nrf2* signalling signature is active in anlotinib‐resistant patient tumours. (A) RT‐qPCR analysis showing elevated miR‐941 levels in tumour tissues from patients resistant to anlotinib compared to sensitive patients. (B) Representative Western blots and quantitative results showing downregulation of *Keap1* and upregulation of *Nrf2* and its downstream targets (*Bcl‐2*, *Mcl‐1*, *Bcl‐xL*, *TNF‐α*, *MMP9*, *MMP27*) in resistant tumours. (C–E) RT‐qPCR analysis confirming upregulation of the mRNA levels of (C) anti‐apoptotic genes (*Bcl‐2*, *Mcl‐1*, *Bcl‐xL*), (D) *TNF‐α*, and (E) MMPs (*MMP9*, *MMP27*) in resistant versus sensitive patient tumours. (F, G) Kaplan–Meier survival curves showing (F) progression‐free survival (PFS) and (G) overall survival (OS) of anlotinib‐treated NSCLC patients stratified by tumour *miR‐941* expression (high vs. low, based on median expression value). Data are presented as mean ± SEM. *p*‐values indicated were calculated by Mann–Whitney *U* tests. ns: *p *> 0.05, *p < 0.05, **p < 0.01.

To bridge our mechanistic findings with clinical relevance, we analysed tumour tissue samples from 14 NSCLC patients, classified into resistant or sensitive groups based on their response to anlotinib treatment. RT‐qPCR analysis confirmed that, compared with the sensitive group, the expression levels of *miR‐941* were markedly higher in tumours from the resistant group (Figure 6A).

More importantly, Western blot analysis of these samples revealed a distinct molecular signature consistent with pathway activation. Resistant tumours with high *miR‐941* expression exhibited downregulation of Keap1 protein and concomitant upregulation of Nrf2. Furthermore, downstream effectors of *Nrf2*—including anti‐apoptotic proteins (Bcl‐2, Mcl‐1), the pro‐proliferative factor TNF‐α, and the pro‐migratory protein MMP9—were consistently elevated in these samples (Figure 6B). This finding was further supported by RT‐qPCR results showing increased mRNA levels of the corresponding genes (Figure 6C–E).

To further evaluate the prognostic significance of *miR‐941* in anlotinib‐treated patients, we performed Kaplan–Meier survival analysis. Based on the median level of miR‐941 in tumours, subjects were categorized into two groups: high‐ or low‐expression group. As shown in Figure 6F,G, patients with high miR‐941 expression (non‐responders) exhibited significantly shorter PFS and OS compared to those with low miR‐941 expression (Responders). These findings demonstrate that elevated *miR‐941* levels correlate with poor clinical outcomes following anlotinib treatment, further supporting its translational relevance as a predictive and prognostic biomarker.

Together, these clinical data provide strong evidence corroborating the signalling cascade identified in our preclinical models, wherein elevated *miR‐941* expression suppresses Keap1, leading to Nrf2 activation and subsequent oncogenic pathway induction.

## 4 DISCUSSION

Building upon our previous clinical observation that anlotinib exhibits significant heterogeneity in efficacy among real‐world NSCLC patients,^14^ this study provides compelling evidence that EV‐derived *miR‐941* plays a pivotal functional role in mediating anlotinib resistance. We demonstrate that elevated levels of EV‐*miR‐941* are not only correlated with clinical resistance but are also functionally capable of driving the resistant phenotype. Mechanistically, *miR‐941* directly targets *Keap1*, leading to activation of the *Nrf2* signalling pathway, which in turn suppresses anlotinib‐induced apoptosis and promotes malignant progression. This robust signalling axis was consistently validated in both mouse models and clinical tumour specimens.

Our findings position *miR‐941* not merely as a correlative biomarker, but as a functional driver of anlotinib resistance. This finding highlights the context‐dependent role of miRNAs, where a single miRNA can be used to execute highly specific functions, in this case conferring resistance to a particular anti‐angiogenic TKI, thereby expanding our understanding of miRNA functional plasticity in cancer. Through gain‐ and loss‐of‐function experiments along with EV‐based transfer assays, we established that *miR‐941* actively induces therapeutic resistance, representing a critical advance in translational understanding. While *miR‐941* has previously been implicated in promoting proliferation in breast cancer^15^ and lung adenocarcinoma,^16^ our work is the first to demonstrate its direct functional role in conferring resistance to a specific therapeutic agent, anlotinib. Moreover, the stability, non‐invasiveness and dynamic monitoring potential of EV‐derived *miR‐941* underscore its promise as an ideal biomarker for serial assessment of emerging resistance.

The *Keap1*–*Nrf2* pathway is a well‐established central hub in mediating resistance to multiple anticancer therapies. Beyond its involvement in anlotinib resistance demonstrated here, this pathway has been extensively implicated in conferring resistance to diverse chemotherapeutic agents such as cisplatin,^17^, ^18^ as well as to various targeted therapies.^19^, ^20^ This broad role underscores the fundamental importance of *Nrf2* activation in cancer cell survival and highlights the potential universality of our findings. A key mechanistic insight from our study is the identification of a miRNA‐mediated, mutation‐independent mechanism of *Nrf2* pathway activation. Unlike canonical genetic alterations such as mutations in *KEAP1* or *NFE2L2* (which encodes *Nrf2*) that lead to constitutive *Nrf2* activation,^21^ *miR‐941*–induced suppression of *Keap1* represents a more dynamic and readily inducible form of regulation. This post‐transcriptional strategy allows tumour cells to rapidly adapt to therapeutic pressure without relying on the slower clonal selection of pre‐existing mutations.^22^ Such plasticity may be particularly relevant for the development of “adaptive resistance,” wherein tumours evade drug effects soon after treatment initiation. While *KEAP1* or *NFE2L2* mutations often occur early in tumorigenesis and provide a constitutive survival advantage,^23^, ^24^ *miR‐941*–driven *Nrf2* activation may be preferentially employed or upregulated in response to specific selective pressures. This non‐mutational activation represents an alternative regulatory mechanism that allows cancer cells to circumvent drug pressure, thereby expanding the repertoire of resistance mechanisms available to them.

Activation of the *Nrf2* pathway exerts multifaceted pro‐oncogenic effects that extend beyond drug resistance. A direct mechanism through which *Nrf2* activation confers anlotinib resistance is the transcriptional upregulation of anti‐apoptotic proteins, particularly members of the *Bcl‐2* family. *Nrf2* has been shown to enhance the expression of *Bcl‐2*, *Bcl‐xL*, and *Mcl‐1*, thereby directly countering the apoptosis‐inducing activity of therapeutics.^25^, ^26^ By shifting the balance toward cell survival, this anti‐apoptotic function enables cancer cells to withstand treatment‐induced stress. Notably, our study further reveals that the *miR‐941*/*Keap1*/*Nrf2* axis also promotes malignant behaviours such as proliferation and migration. *Nrf2* activation led to increased expression of proliferative mediators like *TNF‐α* and metastasis‐associated matrix metalloproteinases including *MMP9*, illustrating that this pathway does not only promotes cell survival but also enhances aggressiveness. This pro‐invasive dimension helps explain the association between high *miR‐941* levels and poorer patient prognosis, as the same molecular mechanism that supports resistance also drives disease progression. The co‐occurrence of resistance and malignant phenotypes under Nrf2 activation confers a significant survival and metastatic advantage to cancer cells.^27^ This dual functionality positions the miR‐941/Keap1/Nrf2 axis not only as a mediator of resistance but as a crucial regulator of tumour progression and drug response. This intrinsic coupling of resistance and aggressiveness provides a molecular explanation for why tumours that become drug‐resistant often emerge with a more malignant phenotype, leading to dismal patient outcomes.

The most immediate clinical implication of our findings lies in the potential of EV‐derived *miR‐941* to serve as a non‐invasive predictive biomarker for anlotinib response. Liquid biopsy‐based detection of circulating EV‐*miR‐941* offers significant advantages, including ease of serial sampling, real‐time monitoring capability and strong correlation with tumour behaviour.^28^ As a functional driver of resistance, *miR‐941* expression may more directly reflect intrinsic tumour drug sensitivity, thereby addressing a critical unmet need in patient stratification. Beyond its role as a biomarker, the *miR‐941*/*Keap1*/*Nrf2* axis presents a promising therapeutic target. Targeting *miR‐941* using anti‐miR oligonucleotide or locked nucleic acid‐based inhibitors could restore *Keap1* expression and prevent *Nrf2* activation, thereby re‐sensitizing tumours to anlotinib. Alternatively, direct inhibition of *Nrf2* signalling with pharmacological agents such as ML385 offers a complementary strategy. Our experimental data using ML385 provide preliminary proof‐of‐concept for this approach. These strategies align with growing efforts to target non‐oncogene dependencies and adaptive resistance mechanisms in cancer therapy.^29^, ^30^ Thus, the translational value of this work is twofold: it proposes a novel biomarker to guide patient selection and highlights a new therapeutic axis whose inhibition may reverse resistance and improve outcomes in anlotinib‐treated NSCLC.

However, the present study has certain limitations. First, the initial hypothesis was generated from a retrospective cohort, which may introduce selection bias. The clinical sample size for mechanistic validation (*n* = 14) remains limited, necessitating future prospective, large‐scale studies to rigorously establish the predictive value and optimal cut‐off for plasma EV‐*miR‐941*. Second, the modest sample size precluded meaningful subgroup analyses to determine whether elevated *miR‐941* expression is preferentially enriched in specific patient populations. Given that anlotinib is often administered in later‐line settings where driver mutation status and tumour immune contexture may influence therapeutic response, future studies with larger, well‐annotated cohorts are warranted to explore whether *miR‐941*‐mediated resistance exhibits distinct clinical associations. Third, while EV purity was confirmed by WB analysis of CD63, TSG101, CD9 and CD81 (Figure 1F), we did not perform nanoparticle tracking analysis or transmission electron microscopy to further characterize EV size distribution and morphology. The absence of these orthogonal characterization methods represents a limitation of the current study. Future investigations will incorporate comprehensive EV characterization to provide more rigorous validation of EV integrity and purity. Moreover, although our genetic rescue experiments confirm the central role of the Keap1‐Nrf2 axis, we cannot exclude the possibility that additional off‐targets of miR‐941 may also contribute to the resistant phenotype. Comprehensive miRNA target profiling or transcriptome‐wide analysis would provide further biological insight. Fourth, although our in vitro findings were validated in both A549 and H1299 cell lines, further validation across a broader panel of genetically diverse NSCLC models is warranted. Subcutaneous xenografts, although informative, do not fully recapitulate the lung tumour microenvironment or metastatic process. Additionally, the therapeutic potential of combination strategies remains unexplored in vivo. Finally, although serum EVs are enriched in tumour‐derived vesicles, their heterogeneous cellular origins preclude definitive attribution to cancer cells alone. Moreover, given that extracellular vesicles are key intermediaries in intercellular communication in the tumour microenvironment, future studies investigating whether EV‐derived *miR‐941* influences the function of stromal components such as tumour‐associated macrophages or cancer‐associated fibroblasts, and whether such interactions contribute to anlotinib resistance, would further broaden the translational impact of our findings.

Future research should focus on the prospective clinical validation of plasma EV‐*miR‐941* as a predictive biomarker. It will also be essential to investigate the function of this axis in orthotopic or patient‐derived xenograft models that better mimic human disease. Exploring the efficacy of *Nrf2* inhibitors in combination with anlotinib represents another critical direction. Beyond anlotinib, it would be worthwhile to examine whether the *miR‐941*/*Keap1*/*Nrf2* axis also mediates resistance to other anti‐angiogenic TKIs or conventional therapies. From a clinical perspective, we propose that plasma EV‐*miR‐941* could serve as a practical biomarker for guiding anlotinib therapy. Specifically, *EV‐miR‐941* levels might be assessed prior to treatment initiation to identify patients with intrinsically elevated expression who may be less likely to benefit from anlotinib monotherapy. In addition, serial monitoring during treatment could help detect the emergence of acquired resistance. For patients with high *EV‐miR‐941* levels, combination strategies targeting the *miR‐941‐Keap1‐Nrf2* axis, such as Nrf2 inhibitors, may represent a rational therapeutic approach, whereas those with low levels may achieve more favourable responses to anlotinib alone. In addition, while our genetic rescue experiments demonstrate that Keap1 is a major functional mediator, future transcriptome‐wide analyses (e.g., RNA‐seq after miR‐941 modulation) will be valuable to comprehensively identify all direct and indirect targets of miR‐941, uncover potential off‐target effects, and further elucidate the broader regulatory network underlying anlotinib resistance. Furthermore, the molecular mechanisms governing the sorting and secretion of miR‐941 into extracellular vesicles remain unexplored. Future studies investigating whether key regulators of EV biogenesis, such as Rab27a, nSMase2, or other components of the ESCRT machinery, modulate the release of EV‐miR‐941 would further elucidate the upstream events that control this resistance axis.

Overall, our study provides the first demonstration that *miR‐941* serves as a key functional mediator of anlotinib resistance in NSCLC through direct targeting of *Keap1* and consequent activation of the *Nrf2* pathway. This discovery expands the current understanding of *miRNA*‐mediated drug resistance by revealing a previously unrecognized, context‐dependent role for *miR‐941* that is both therapeutically relevant and mechanistically distinct from previously reported miRNA functions. These findings not only elucidate a novel resistance mechanism but also highlight EV‐*miR‐941* as a promising non‐invasive biomarker and therapeutic target, providing an actionable biomarker for precision therapy in advanced NSCLC.

## AUTHOR CONTRIBUTIONS

Aimi Huang and Xiaoqi Li were responsible for the research, contributed to data collection and prepared the draft. Jinjing Xia and Erpeng Wu contributed to the study design and performed the statistical analysis. Menglan Hao and Weimin Wang contributed to data collection, analysis, or interpretation and drafted the manuscript.

## CONFLICT OF INTEREST STATEMENT

The authors declare no conflicts of interest.

## CONSENT

Informed consent was obtained from all participants, who were fully informed of the study protocol.

## ETHICS STATEMENT

The present research has been granted approval by the Ethics Committee of Shanghai Chest Hospital (IS22010). The authors affirm that all research methods comply with relevant guidelines.

## Supporting information



(A) Cell viability of H1299 cells transfected with *miR‐941* mimic, *miR‐941* mutant (mut), or negative control (NC) and treated with anlotinib (10 µM). Data are presented as % ATP level.
(B) Western blot analysis of Keap1 and Nrf2 protein levels in H1299 cells following transfection with *miR‐941* mimic, *miR‐941* mut, or NC. GAPDH served as loading control.
(C) Nuclear‐cytoplasmic fractionation and Western blot analysis of Nrf2 subcellular localization in H1299 cells transfected with *miR‐941* mimic or NC. GAPDH and Histone H3 served as cytoplasmic and nuclear loading controls, respectively. Quantification represents three independent experiments.
Error bars indicate mean ± SD. p‐values were calculated by Student's *t*‐test (unpaired). ns: *p* > 0.05, ***p < 0.001.

(A) Quantification of apoptotic cell percentage in H1299 cells.
(B) Western blot analysis of cleaved PARP and cleaved Caspase‐3 in H1299 cells. Error bars represent standard deviation (*n* = 3). *p*‐values were calculated by Student's *t*‐test (unpaired). *p < 0.05, ***p < 0.001.

(A) Western blot analysis of MMP9 and MMP27 in H1299 cells transfected with *miR‐941* mimic, *miR‐941* mut, or NC.
(B) Western blot analysis of Bcl‐xL, Bcl‐2 and Mcl‐1 in H1299 cells transfected with *miR‐941* mimic, *miR‐941* mut, or NC.
(C) Representative images of wound healing assays in H1299 cells at 0 h (D0) and 24 h (D2). Scale bar, 200 µm.
(D) Quantification of relative wound area in H1299 cells.
Error bars represent standard deviation (*n* = 3). *p*‐values were calculated by Student's *t*‐test (unpaired). ***p < 0.001.

(A) Representative images of Transwell invasion assays in H1299 cells. Scale bar, 100 µm.
(B) Quantification of invaded cell numbers per field.
Error bars represent standard deviation (*n* = 3). *p*‐values were calculated by Student's *t*‐test (unpaired). ***p < 0.001.

Supporting Information

## Data Availability

This work includes all data that are generated or analysed during the course of the research.
