# Supplementary figures and images for: miR‐941 in extracellular vesicles confers anlotinib resistance via Keap1/Nrf2 axis and represents a therapeutic target in non‐small cell lung cancer
Source: Clin Transl Med. 2026 Jun 15;16(6):e70721. doi: 10.1002/ctm2.70721 (PMC13269834; doi:10.1002/ctm2.70721)

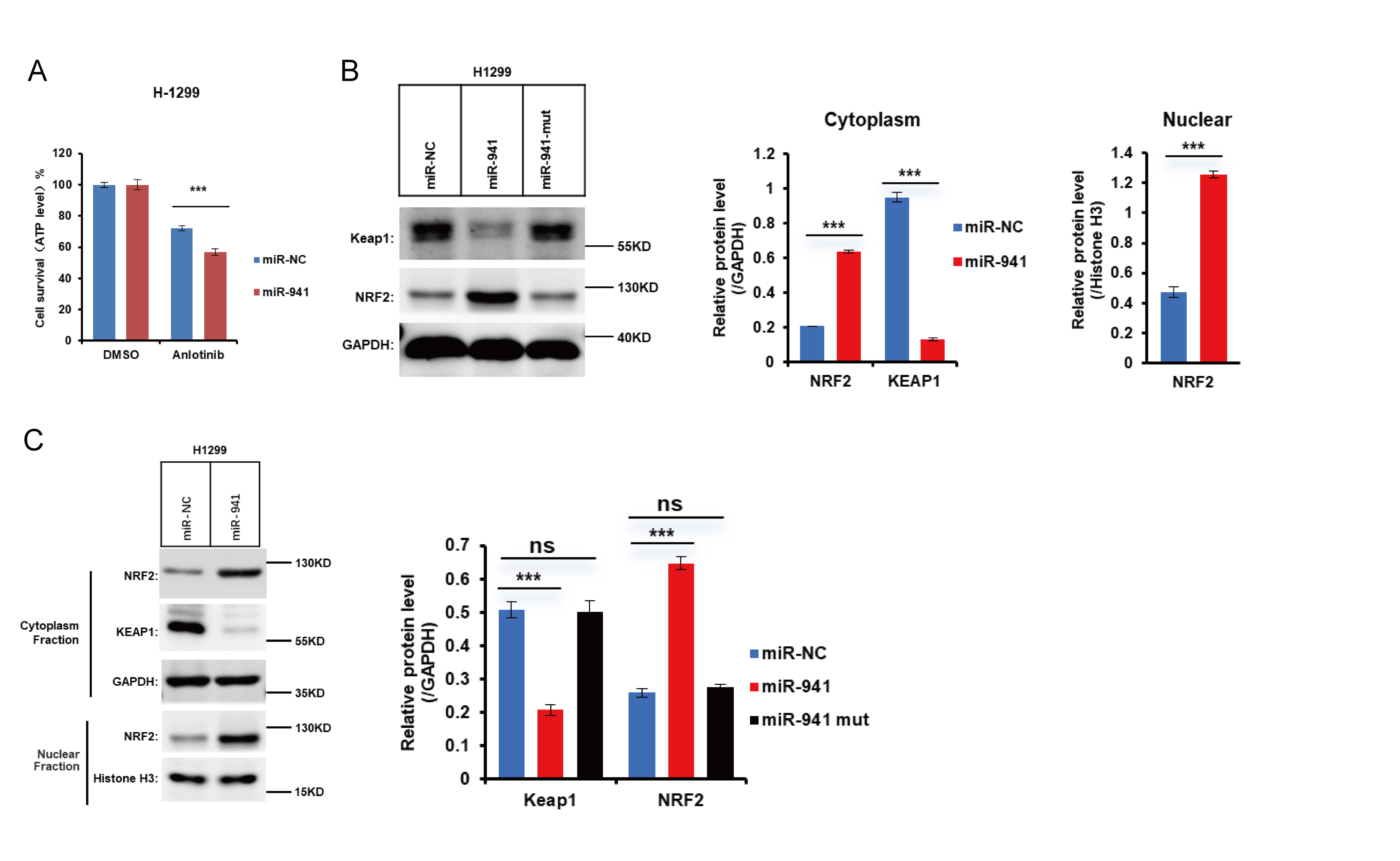

Supplement: Supplementary file 1 — (A) Cell viability of H1299 cells transfected with miR‐941 mimic, miR‐941 mutant (mut), or negative control (NC) and treated with anlotinib (10 µM). Data are presented as % ATP level. (B) Western blot analysis of Keap1 and Nrf2 protein levels in H1299 cells following transfection with miR‐941 mimic, miR‐941 mut, or NC. GAPDH served as loading control. (C) Nuclear‐cytoplasmic fractionation and Western blot analysis of Nrf2 subcellular localization in H1299 cells transfected with miR‐941 mimic or NC. GAPDH and Histone H3 served as cytoplasmic and nuclear loading controls, respectively. Quantification represents three independent experiments. Error bars indicate mean ± SD. p‐values were calculated by Student's t‐test (unpaired). ns: p > 0.05, ***p < 0.001. [file CTM2-16-e70721-s004.tif]

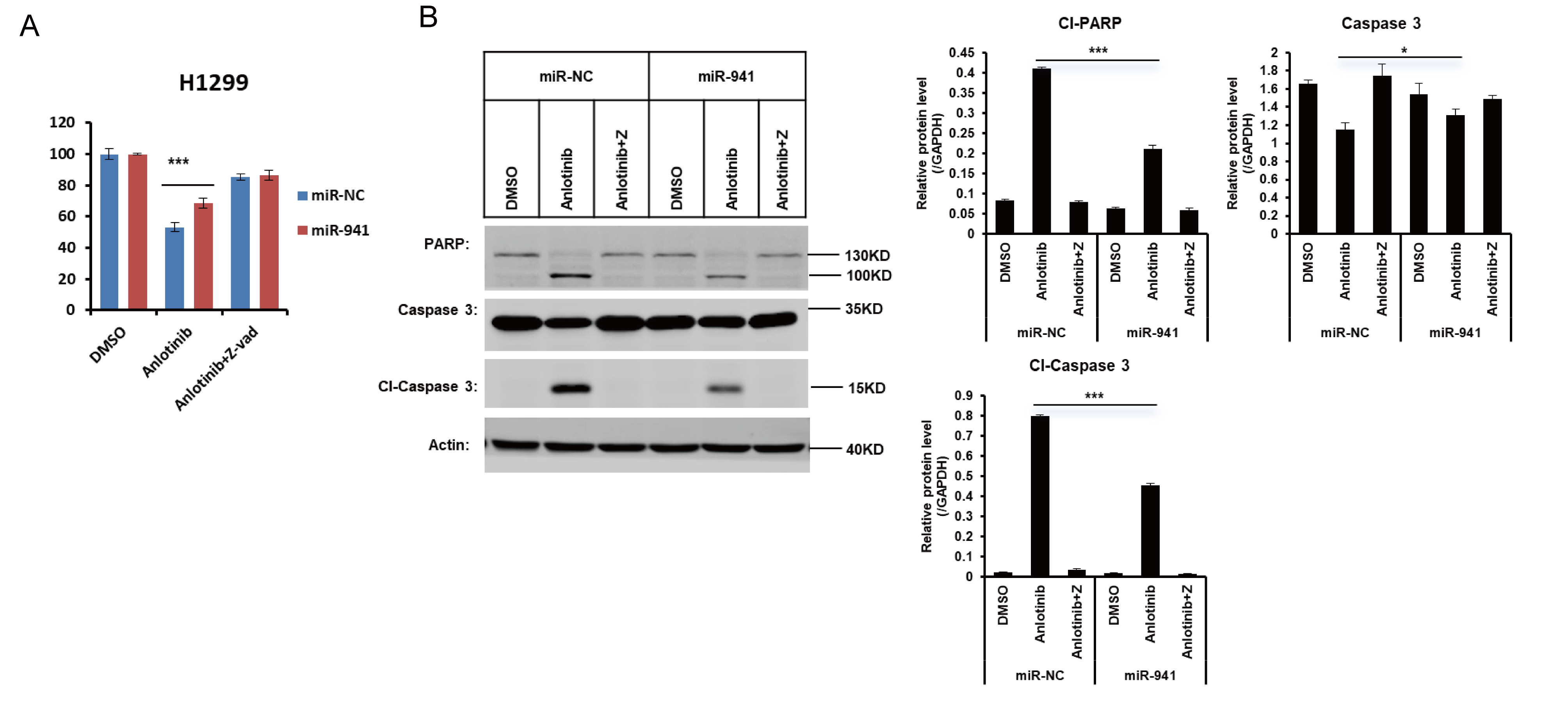

Supplement: Supplementary file 2 — (A) Quantification of apoptotic cell percentage in H1299 cells. (B) Western blot analysis of cleaved PARP and cleaved Caspase‐3 in H1299 cells. Error bars represent standard deviation (n = 3). p‐values were calculated by Student's t‐test (unpaired). *p < 0.05, ***p < 0.001. [file CTM2-16-e70721-s003.tif]

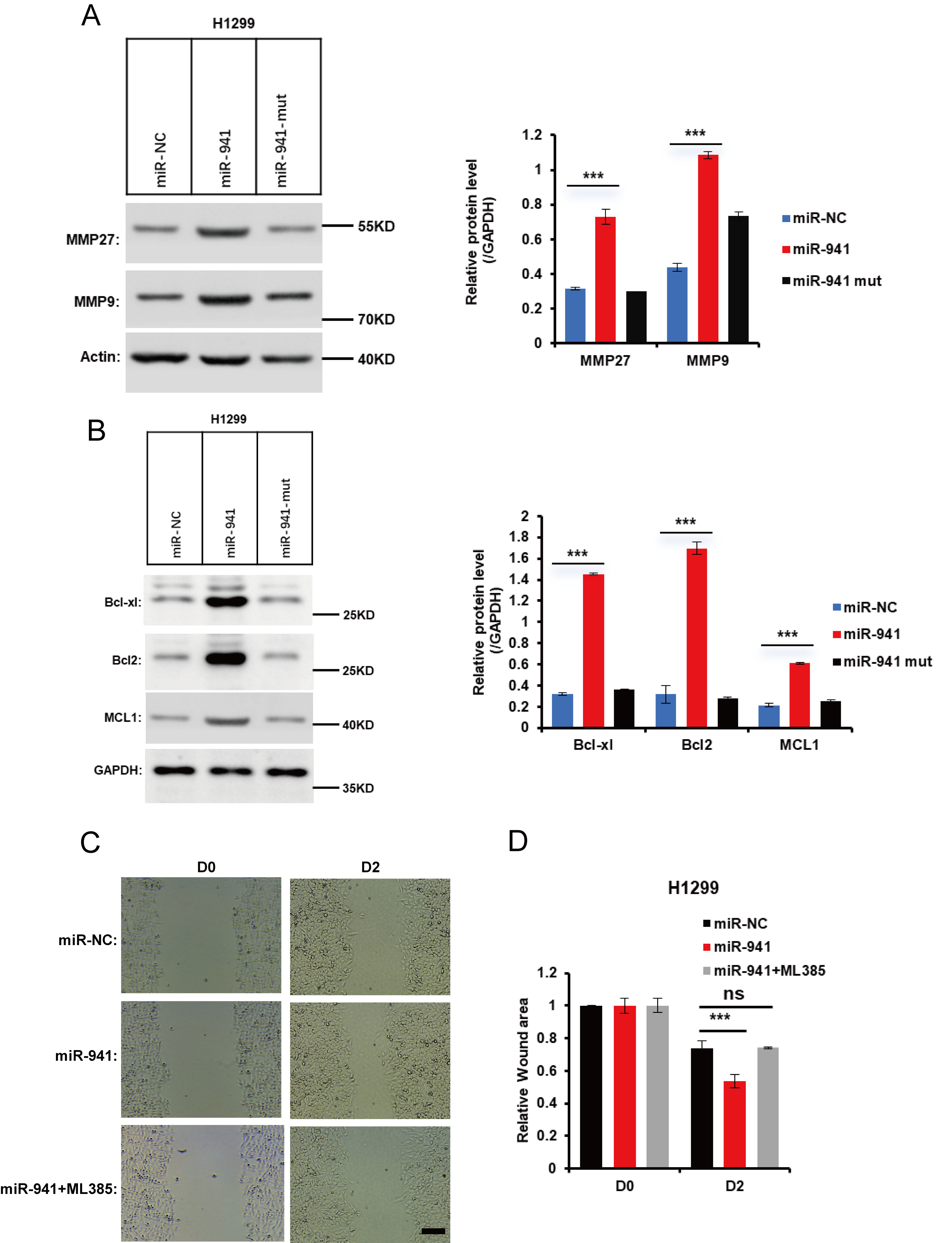

Supplement: Supplementary file 3 — (A) Western blot analysis of MMP9 and MMP27 in H1299 cells transfected with miR‐941 mimic, miR‐941 mut, or NC. (B) Western blot analysis of Bcl‐xL, Bcl‐2 and Mcl‐1 in H1299 cells transfected with miR‐941 mimic, miR‐941 mut, or NC. (C) Representative images of wound healing assays in H1299 cells at 0 h (D0) and 24 h (D2). Scale bar, 200 µm. (D) Quantification of relative wound area in H1299 cells. Error bars represent standard deviation (n = 3). p‐values were calculated by Student's t‐test (unpaired). ***p < 0.001. [file CTM2-16-e70721-s005.tif]

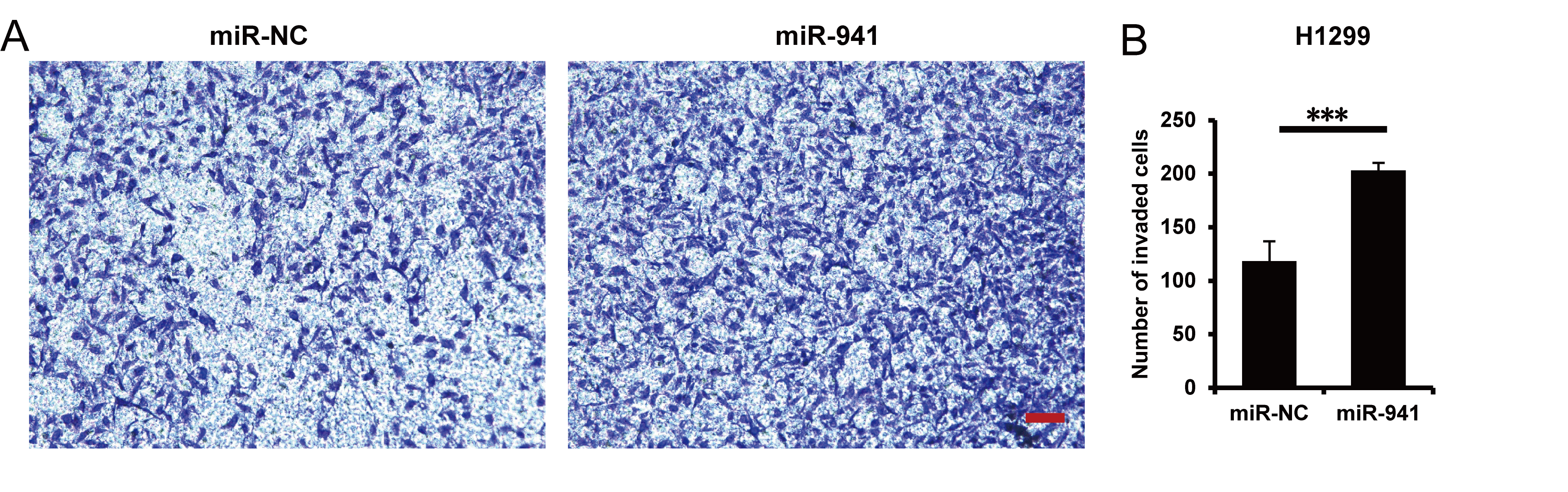

Supplement: Supplementary file 4 — (A) Representative images of Transwell invasion assays in H1299 cells. Scale bar, 100 µm. (B) Quantification of invaded cell numbers per field. Error bars represent standard deviation (n = 3). p‐values were calculated by Student's t‐test (unpaired). ***p < 0.001. [file CTM2-16-e70721-s002.tif]
