# Supplementary material for: miR‐941 in extracellular vesicles confers anlotinib resistance via Keap1/Nrf2 axis and represents a therapeutic target in non‐small cell lung cancer
Source: Clin Transl Med. 2026 Jun 15;16(6):e70721. doi: 10.1002/ctm2.70721 (PMC13269834; doi:10.1002/ctm2.70721)
Supplement: Supplementary file 5 — Supporting Information [file CTM2-16-e70721-s001.docx]

**Table S1. Baseline Characteristics and Survival Outcomes in Non-Responder Group and Responder Cohort**

| Characteristic |  | Non-Responder Group(n=6) | Responder Cohort(n=8) | *P*-value | HR (95% CI) |
| --- | --- | --- | --- | --- | --- |
| **Gender, n (%)** | Male | 4 (66.7) | 6 (75.0) | 1.000 |  |
|  | Female | 2 (33.3) | 2 (25.0) |  |  |
| **Age, years** | Median | 63 | 64 |  |  |
| **Histological Type, n (%)** | Adenocarcinoma | 3 (50.0) | 7 (87.5) | 0.348 |  |
|  | Squamous cell carcinoma | 3 (50.0) | 1 (12.5) |  |  |
| **Line of Therapy, n (%)** | Second-line | 4 (66.7) | 1 (12.5) | 0.103 |  |
|  | Third-line | 2 (33.3) | 6 (75.0) |  |  |
|  | Later-line | 0 (0.0) | 1 (12.5) |  |  |
| **Prior Treatment, n (%)** | Chemotherapy | 4 (66.7) | 3 (37.5) | 0.078 |  |
|  | Targeted Therapy | 1 (16.7) | 2 (25.0) |  |  |
|  | Chemotherapy + Targeted | 1 (16.7) | 3 (37.5) |  |  |
| **PFS, months** | Median | 1.35 | 4.62 | **<0.001** | **4.89 (2.13-11.25)** |
| **OS, months** | Median | 4.72 | 14.39 | **<0.01** | **5.62 (2.45-12.88)** |

Abbreviations: PFS, progression-free survival; OS, overall survival; HR, hazard ratio; CI, confidence interval.

**Table S2. List of sgRNA sequences for CRISPR/Cas9 knockout**

| **Target Gene** | **sgRNA Sequence (5' to 3')** |
| --- | --- |
| *Keap1* sgRNA | GTGTGTCCTCCACGTCATGAA |
| *Nrf2* sgRNA | GGACATTGAGCAAGTTTGGG |

**Supplementary materials**

**Figure S1. Validation of the miR-941/Keap1/Nrf2 axis in H1299 NSCLC cells.**

(A) Cell viability of H1299 cells transfected with *miR-941* mimic, *miR-941* mutant (mut), or negative control (NC) and treated with anlotinib (10 μM). Data are presented as % ATP level.

(B) Western blot analysis of Keap1 and Nrf2 protein levels in H1299 cells following transfection with *miR-941* mimic, *miR-941* mut, or NC. GAPDH served as loading control.

(C) Nuclear-cytoplasmic fractionation and Western blot analysis of Nrf2 subcellular localization in H1299 cells transfected with *miR-941* mimic or NC. GAPDH and Histone H3 served as cytoplasmic and nuclear loading controls, respectively. Quantification represents three independent experiments.

Error bars indicate mean ± SD. p-values were calculated by Student's *t*-test (unpaired). ns: *p* > 0.05, ****p* < 0.001.

**Figure S2. *miR-941* suppresses anlotinib-induced apoptosis in H1299 cells.**

(A) Quantification of apoptotic cell percentage in H1299 cells.

(B) Western blot analysis of cleaved PARP and cleaved Caspase-3 in H1299 cells. Error bars represent standard deviation (n=3). *p*-values were calculated by Student's *t*-test (unpaired). **p* < 0.05, ****p* < 0.001.

**Figure S3. *miR-941* promotes migration and upregulates anti-apoptotic proteins in H1299 cells.**

(A) Western blot analysis of MMP9 and MMP27 in H1299 cells transfected with *miR-941* mimic, *miR-941* mut, or NC.

(B) Western blot analysis of Bcl-xL, Bcl-2 and Mcl-1 in H1299 cells transfected with *miR-941* mimic, *miR-941* mut, or NC.

(C) Representative images of wound healing assays in H1299 cells at 0 h (D0) and 24 h (D2). Scale bar, 200 μm.

(D) Quantification of relative wound area in H1299 cells.

Error bars represent standard deviation (n=3). *p*-values were calculated by Student's *t*-test (unpaired). ****p* <0.001.

**Figure S4. *miR-941* enhances invasive capacity of H1299 cells.**

(A) Representative images of Transwell invasion assays in H1299 cells. Scale bar, 100 μm.

(B) Quantification of invaded cell numbers per field.

Error bars represent standard deviation (n=3). *p*-values were calculated by Student's *t*-test (unpaired). ****p* < 0.001.
